# Differential brain neuroimmune profiles and altered immune balance in autism spectrum disorder compared with psychotic disorder and neurotypical controls

**DOI:** 10.21203/rs.3.rs-9500684/v1

**Published:** 2026-05-20

**Authors:** Hadley Osman, Elizabeth Breece, Paul Ashwood

**Affiliations:** University of California Davis; University of California Davis; University of California Davis

**Keywords:** Psychotic Disorder, Psychosis, autism, autism spectrum disorders, ASD, cytokines, IL-1, brain, neuroinflammation, STG region, TGFβ1, regulation, inflammatory, anti-inflammatory

## Abstract

Alterations in immune and neuroinflammatory processes are evident in neurodevelopmental disorders, including autism (ASD) and psychotic disorders. While prior studies using cerebral spinal fluid and serum have identified differences in cytokine levels in ASD or psychotic disorders when compared with controls, specific changes in postmortem human brain tissue remains poorly characterized, making cross-disorder comparisons uncertain. The superior temporal gyrus (STG), involved in social communication and sensory processing, is affected in both conditions, making it a relevant region for investigating immune-related differences. This study aimed to compare neuroimmune profiles in ASD and psychotic disorders. STG tissue was analyzed from 72 postmortem cases: ASD (n = 17), psychotic disorders (n = 26), and neurotypical controls (n = 29). Fresh-frozen samples were collected using biopsy punches, and cytokines, chemokines, and growth factors were quantified using multiplexed bead immunoassays. In ASD cases, there were significantly increased levels of inflammatory cytokines IL-1α, IL-8, G-CSF, M-CSF and GROa compared to both controls and psychotic disorder. In contrast, there was a significant decrease in IFNα2 and IL-4 cytokine levels in ASD compared to both controls and psychotic disorders. In the psychotic disorder group, there were increases in inflammatory chemokines eotaxin, fractalkine and RANTES compared to ASD and controls. Notably, there was a concomitant rise in regulatory cytokines TGFβ1 and IL-10 in psychotic disorder suggestive of compensatory response that was not observed in ASD. Overall, ASD presented a distinctive STG cytokine profile with an increased pro-inflammatory cytokine levels and reduced immune regulatory balance, whereas psychotic disorder exhibited significant variations in chemokines and growth factors but incorporating greater immune regulation. These data highlights that unique neuroimmune pathways occur in different neurodevelopmental disorders offering insights into potential therapeutic targets tailored to each condition.

## Introduction

Autism spectrum disorders (ASD) and psychotic disorders are common mental health conditions that have increased in incidence over the last 10 years. While there is evidence for multiple genetic and environmental factors contributing to these disorders, their precise etiology and pathophysiology remain unknown (Coury et al., 2023; Davies et al., 2020; Hughes et al., 2020; Hughes et al., 2018). One hypothesis is that immune dysfunction plays a significant role in their development. Supporting this, findings from both epidemiological studies and animal models suggest that cytokine imbalances can disturb neurodevelopment and/or chronically impair brain function (Han et al., 2021; Hughes et al., 2023). Cytokines and their receptors are expressed by neurons and glia throughout development, influencing a range of physiological processes including neural progenitor differentiation, neuronal migration, axonal guidance, neurotrophin secretion, long-term potentiation and synaptic plasticity, which affect cognitive function, learning, and memory (Deverman & Patterson, 2009; Zengeler & Lukens, 2021). Notably, cytokines exert effects within a narrow homeostatic range and any deviations above or below physiological levels can lead to impairments in function and connectivity.

Numerous studies have examined cytokine levels in plasma, serum, and cerebrospinal fluid (CSF) to characterize immune profiles in neurodevelopmental and psychiatric disorders. Meta-analyses highlight elevated levels of cytokines in peripheral blood in individuals with psychotic disorders and those with ASD relative to healthy, typically developing, unaffected controls individuals (Halstead et al., 2023; Saghazadeh et al., 2019). In non-medicated individuals with first episode psychosis (FEP), interleukin (IL)-1β, IL-6, IL-17, interferon (IFN)-γ, tumor necrosis factor (TNF)-α and soluble IL-2 receptor (sIL-2R) were significantly elevated (Upthegrove et al., 2014; Pillinger et al., 2019). In addition, the regulatory cytokine, transforming growth factor (TGF)-β1 was significantly increased in FEP (Pillinger et al., 2019). Potential state markers during acute exacerbations consisted of increased IL-1, IL-6, and TGFβ1 (Potvin et al., 2008; Miller et al., 2011). Furthermore, a meta-analysis of CSF cytokines found increases in IL-1, IL-6 and IL-8 in schizophrenia (Wang et al., 2018). In ASD, meta-analyses of plasma revealed increased inflammatory cytokines IL-1β, IL-6, IL-8, IFNg, and monocyte chemoattractant protein (MCP)-1; however, there were lower levels of TGFβ1 (Saghazadeh et al., 2019, Masi et al., 2015). Despite these findings, there remains a knowledge gap of whether plasma and CSF cytokine levels reflect neuroinflammatory processes in the brain itself.

The superior temporal gyrus (STG) region modulates sensory integration and social emotional processes, thereby playing a critical role in integrating a breadth of information to provide meaning of the surrounding world (Jou et al., 2010). Structural and functional imaging studies have long implicated the STG in ASD (Jou et al., 2010, Amaral et al., 2008, Gros et al., 2024) and schizophrenia (Kasai et al., 2003). Transcriptomic analyses of STG postmortem brain samples from individuals with ASD revealed neuron-specific changes in synaptic signaling, heat shock protein-related pathways, RNA splicing and upregulated AP-1-mediated neuroinflammation and insulin/IGF-1 signaling pathways compared with healthy controls (Zhang et al., 2023). Mechanistic modeling suggested a direct link between inflammation and ASD in neurons, and prioritized inflammation-associated genes for future study (Zhang et al., 2023). However, these gene expression studies did not identify specific cytokine mediators that could take part in the neuroimmune process in the STG.

Due to the potential biological and therapeutic importance of cytokines in neurodevelopmental disorders there is a need to determine the overlap or divergence in cytokines profiles in the brain in these conditions. This study aimed to identify neuroinflammatory changes in individuals with ASD and psychotic disorders by quantifying levels of pro-inflammatory cytokines, chemokines and growth factors in the STG. In addition, we assessed the balance between pro-inflammatory and anti-inflammatory cytokines, to identify unique biomarker signatures altered in ASD and/or psychotic disorders.

## Methods

### Tissue Acquisition and Processing

Fresh frozen superior temporal gyrus (STG) specimens from 72 individuals, including controls (CTRL, n = 29, 24M/5F), autism spectrum disorder (ASD, n = 17, 14M/3F), and psychotic disordere (n = 26, 19M/7F) cohorts (supplemental table 1) were obtained from the NIH NeuroBioBank (University of Maryland) and Brain Endowment for Mental Health (BEMH, University of California, Davis). Tissue and clinical data collection procedures were approved by the institutional review board (IRB) and Human and Anatomical Specimens Tissue Oversight Committee (HASTOC) at the University of California, Davis School of Medicine. Informed consent was obtained from next-of kin at the time of brain tissue collection for follow up to collect donor clinical information to confirm diagnoses. All methods were carried out in accordance with guidelines and regulations of the institutional review boards and approved by at the University of California, Davis. Categorical diagnoses were determined by a combination of medical records, next-of-kin (NOK) and family interviews/questionnaires, and standardized assessments, including modified versions of the Autism Diagnostic Interview-revised (ADI-R) for ASD and the Structured Clinical Interview for DSM-5 for psychotic disorders. Psychotic disorder consisted of schizophrenia, schizoaffective disorder, and primary psychosis. Controls were identified as having no recorded diagnosis of a major neurological or psychiatric disorder, and no other medical condition having a major impact on the brain. Mental health diagnoses for psychotic disorders, ASD and CTRL were confirmed by a mental health trained clinician via the Structured Clinical Interview for DSM-5 (First et al., 2015). For more information on ASD and control procedures, see: https://www.autismbrainnet.org/donor-criteria/

Whole brain specimens collected through BEMH were bisected into separate hemispheres upon intake. One Hemispheres was coronally blocked into 1 cm thick slabs, snap frozen in liquid nitrogen vapor, and stored at −80 °C until use. Samples for multiplex bead assays were collected from fresh frozen tissue blocks using 2mm tissue biopsy punches at a depth of 2 to 3 mm from STG, according to the anatomical definition supplied by the Atlas of the Human Brain” fourth edition (Mai et al., 2015).These samples were mechanically homogenized using a Biomasher II Micro Tissue Homogenizer (Kimble) in the presence of 200ul extraction medium (0.1% Triton x, COMPLETE Protease inhibitor [Roche, 4693116001], and sterile PBS). Homogenates were centrifuged for 10 minutes at 2000rpm and then the supernatant was separated into aliquots to be utilized for multiplex panels and protein normalization.

## Cytokine measures

Brain STG concentrations of 48 cytokines and chemokines were measured using a Luminex Multiplex magnetic bead assay (48-plex Human Cytokine/Chemokine/Growth Factor Panel A, Millipore Sigma, cat no HCYTA-60K-PXBK48) and an additional kit was used to measure active TGFβ1 (Millipore Sigma, TGFBMAG-64K-03). Three samples (2 CTRL, 1 Psychosis) had insufficient volume for both multiplex assays and were only evaluated by the 48-plex panel. 25 μl of the sample lysate was loaded onto the plate, and the assay was run according to the manufacturer’s directions. The sample was incubated with antibody-conjugated fluorescent beads overnight at 4°C. Following incubation, beads were incubated with detection antibodies and then streptavidin-PE. Sample cytokine concentrations were measured using a flow-based Luminex array system (Bio-Plex 200; Bio-Rad Laboratories, Inc.) and calculated using a standard curve of known cytokine ranges. The minimum level of detection for each cytokine and chemokine are as follows (pg/mL): sCD40L: 5.65, EGF: 3.20, eotaxin: 3.08, FGF-2: 22.30, FLT-3L: 0.84, fractalkine: 29.75, G-CSF: 3.76, GM-CSF: 1.55, GROα: 1.05, IFNα2: 6.56, IFNγ: 0.86, IL-1α: 2.27, IL-1β: 0.52, IL-1RA: 1.29, IL-2: 0.28, IL-3: 0.28, IL-4: 0.20, IL-5: 0.17, IL-6: 0.14, IL-7: 0.14, IL-8: 0.52, IL-9: 3.05, IL-10: 0.91, IL-12 (p40): 3.24, IL-12 (p70): 0.88, IL-13: 2.58, IL-15: 0.74, IL-17A: 0.71, IL-17E/IL-25: 19.77, IL-17F: 28.63, IL-18: 0.53, IL-22: 12.68, IL-27: 50.78, IP-10: 2.13, MCP-1: 3.05, MCP-3: 8.61, M-CSF: 31.95, MDC: 0.42, MIG: 3.98, MIP-1α: 3.82, MIP-1β: 0.37, PDGF-AA: 10.33, PDGF-AB/BB: 16.39, RANTES: 1.58, TGFα: 0.97, TNFα: 5.39, TNFβ: 0.80, VEG-F: 0.98. A separate TGFβ−1 assay was performed to determine active-TGFβ1 levels. TGFβ1 requires an additional acidification step to convert latent TGFβ1 to its active form by incubating 25 μl of sample with 10μl of 1 N HCL(Fisher Scientific; Pittsburg, PA) for 30 minutes, followed by the addition of 10 μl of 1.2 N NaOH (Fisher Scientific; Pittsburg, PA) with 0.5 M HEPES (Sigma-Aldrich; St. Louis, MO) for neutralization. The minimum level of detection for TGFβ1 was 6.0 pg/ml. A bicinchoninic acid assay (BCA; ThermoScientific, Rockford, IL) was used to determine total protein for normalization of cytokine/chemokine levels against protein variation.

## Statistical evaluations

Descriptive statistics (frequencies, means, standard deviations, medians, quartiles) were used to summarize the cytokine/chemokine concentrations. The balance of pro- versus anti-inflammatory cytokines in the STG was determined by calculating the ratio of cytokine relative to TGFβ1. Only analytes with a detection rate of > 60% were included in analysis. Cytokines/chemokines that fell below minimum level of detection (MLD) were assigned with MLD/2. A Shapiro-Wilk test was used to assess the normality of the cytokine/chemokine data set and the test indicated non-normality of the data in the majority of analytes (*p* < 0.05). The Kruskal–Wallis rank sum test used to compare non-parametric data between groups and an ANOVA test was used for analysis parametric data, with multiple comparison correction (Sidak) used. Corrected p-values < 0.05 were considered statistically different. Data are expressed as median values (interquartile ranges).

## Results

### Cytokine levels in the STG region differed in individuals with ASD compared to CTRL.

**Figure 1:**
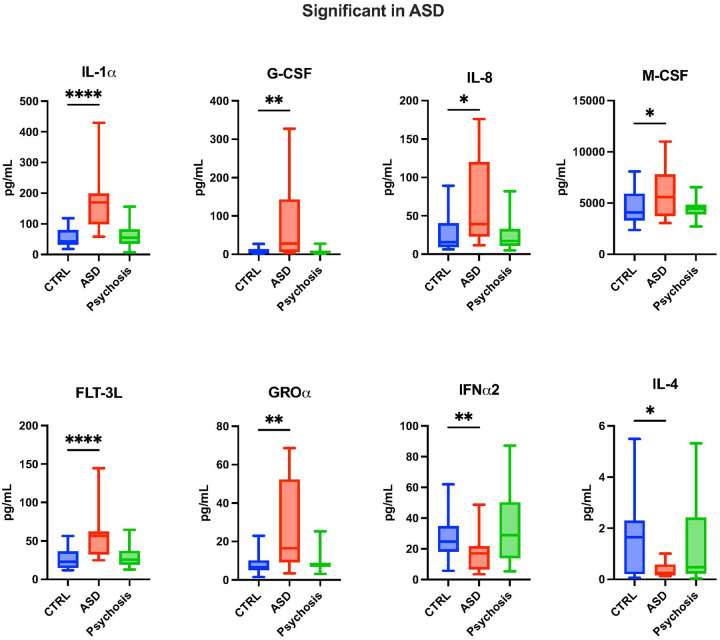
Differential cytokine levels in the STG brain region that are altered in ASD group compared to controls. When compared to the control group (CTRL), IL-1 (*p* < 0.0001), G-CSF (*p* = 0.008), IL-8 (*p* = 0.0155), M-CSF (*p* = 0.0469), FLT-3L (*p* < 0.0001), and GRO (*p* = 0.0019) were significantly elevated. IFN 2 and IL-4 were significantly decreased in the ASD group compared to controls. Significance is represented as: *p<0.05, **p<0.01, ***p<0.001, ****p<0.0001.

We first sought to determine if cytokine levels differed in the STG region between ASD, psychotic disorder and CTRL groups. STG protein levels of 17 cytokines/chemokines mediators were not measured with sufficient accuracy (> 60% of samples) and reproducibility above the detection limit and were excluded from further analyses, these included; sCD40L, GM-CSF, IFNγ, IL1β, IL-2, IL-3, IL-7, IL-12 (p40), IL-13, IL-17A, IL-17E, IL-22, IL-27, MIP-1α, TNFβ, PDGF AA/BB, TNFα. Of the remaining analytes, there were unique profiles observed in ASD compared with controls and psychotic disorders. The ASD group had significantly higher levels of IL-1α (p < 0.0001), IL-18 (p < 0.0001) FLT-3L (p < 0.0001), GROa (p = 0.0019), G-CSF (p = 0.0080), IL-8 (p = 0.015), M-CSF (p = 0.046), compared with CTRL (Fig. 1, Table 1). Conversely, there were lower levels of IFNα2 (p = 0.0065), IL-4 (p = 0.016), and RANTES (p = 0.0035) in ASD compared to CTRL (Fig. 1). Compared with psychotic disorders, there were increased levels of IL-1α (p < 0.0001), IL-18 (p < 0.0001), FLT-3L (p < 0.0001), G-CSF (p = 0.0063), GROa (p = 0.0019), IL-6 (p = 0.0088), IL-8 (0.0094), and MIP-1β (p = 0.0281). Levels of IFNα2 (p = 0.0093) and IL-4 (0.0194) were decreased in ASD compared with psychotic disorders (Table 1; supplemental Table 2).

### Altered cytokine levels in the STG in individuals with psychotic disorders compared with CTRL.

**Figure 2:**
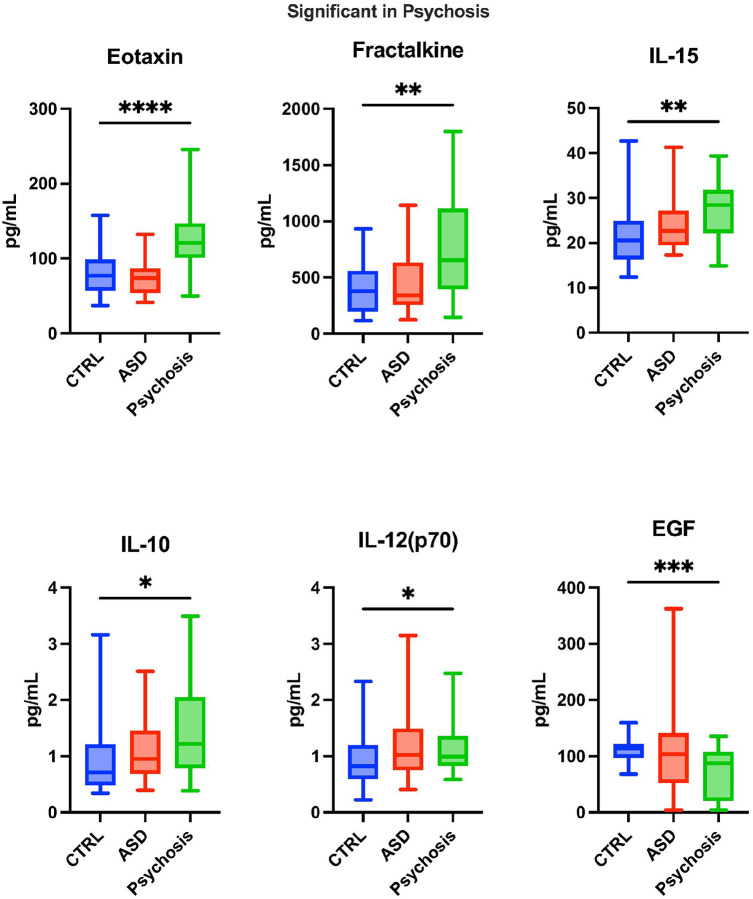
Differential cytokine levels in STG region ltered in psychosis group compared with controls. When compared to the control (CTRL) group, eotaxin (*p* < 0.0001), fractalkine (*p* = 0.002), IL-15 (*p* = 0.0013), IL-10 (*p* = 0.0214), and IL-12 (p70) (*p* = 0.0493) are significantly elevated in the psychosis group. EGF was significantly decreased in the psychosis group when compared with controls (*p* = 0.0009). Significance is represented as: *p<0.05, **p<0.01, ***p<0.001, ****p<0.0001.

In individuals with psychotic disorders, levels of eotaxin (p < 0.0001), IL-15 (p = 0.0013), fractalkine (p = 0.002), IL-10 (p = 0.021), active TGFβ1 (p = 0.0063), and IL-12p70 (p = 0.0493) were increased in the STG compared with CTRL (Fig. 2). Compared with ASD, the levels of eotaxin (p < 0.0001), fractalkine (p = 0.0165), and active TGFβ1 (p = 0.0148) were increased in psychotic disorders (Table 1). EGF levels were significantly decreased in psychotic disorder compared with CTRL and ASD (p = 0.016) (Fig. 2, Table 1)

### Cytokines levels that were differentially represented in both ASD and psychotic disorders.

**Figure 3:**
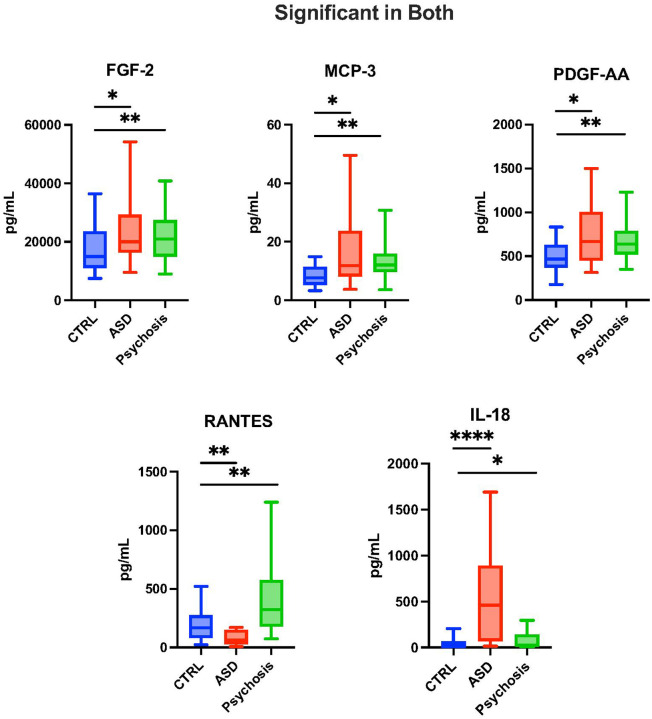
Differential cytokine levels that are altered in STG brain region in both ASD and psychosis groups compared with controls. In both the ASD and psychosis groups, FGF-2 (ASD *p* = 0.0469; psychosis *p* = 0.0178), MCP-3 (ASD *p* = 0.0104; psychosis *p* = 0.0053), PDGF-AA (ASD *p* = 0.02; psychosis *p* = 0.0014), and IL-18 (ASD *p*<0.0001; psychosis *p* = 0.0416) are significantly elevated. RANTES was significantly decreased in ASD (*p* = 0.0035) but elevated in psychosis (*p* = 0.005). Significance is represented as: *p<0.05, **p<0.01, ***p<0.001, ****p<0.0001.

There were additional cytokines that were significantly increased in both ASD and psychotic disorders compared with CTRL (Fig. 3; Table 1), including FGF-2 (ASD p = 0.0469; psychoses p = 0.0178), MCP-3 (ASD p = 0.0104; psychotic disorders p = 0.0053), PDGF-AA (ASD p = 0.0200; psychotic disorders p = 0.0014). Interestingly, a divergent pattern occurred with the chemokine RANTES which was increased in psychotic disorders compared to CTRL (p = 0.005) but decreased in ASD compared to CTRL (p = 0.0035) (Fig. 1; Fig. 2). Moreover, IL-18 levels were decreased in psychotic disorders versus controls (p = 0.0416) but, as mentioned above, increased in ASD.

## Balance of pro- versus anti-inflammatory cytokines in the STG

Imbalance between inflammatory signals and regulatory signals produced by TGFβ1 have been examined previously by calculating ratios of cytokines divided by TGFβ1 as the denominator. In ASD, there was a pattern towards an increased inflammatory profile with higher IL-1/TGFβ1 (p = 0.0003), IL-18/TGFβ1 (p < 0.0001), FLT-3L/TGFβ1 (p = 0.0143) IP-10/TGFβ1 (p = 0.0049), and G- CSF/TGFβ1 (p = 0.0143) ratios compared with CTRL (Table 2). With a similar pattern of statistical significant differences between ASD vs. psychotic disorder for these cytokine ratios (Table 2). In contrast, many ratios were decreased in psychotic disorder for EGF/TGFβ1 (p = 0.0159), FLT3L/TGFβ1 (0.0249), G-CSF/TGFβ1 (p = 0.0208), GRO-α/TGFβ1 (p = 0.0077), IL-1RA/TGFβ1 (p = 0.0046), IL-6/TGFβ1 (p = 0.0071), IL-8/TGFβ1 (p = 0.0090), IL-9/TGFβ1 (p = 0.0264), and CXCL9/TGFβ1 (p = 0.0002), compared to CTRL suggesting the balance of signals is less inflammatory or at least these signals are partially more controlled by regulatory anti-inflammatory TGFβ1 in psychotic disorders (Table 2; Supplemental Table 3).

## Discussion

Glial cells and neurons produce and respond to a diverse array of cytokines that influence their function and phenotype (Deverman et al., 2009; Zengeler et al., 2021). Current dogma suggests a tight physiological range for cytokines to maintain proper brain development and function with deviations either side of this range resulting in altered brain plasticity and behavior. Cytokines, act on neural progenitors to alter neuronal populations, migration, and brain development. They also alter synapse formation to either modulate the expression and function of synaptogenesis proteins or to activate signaling pathways that destabilize or eliminate synapses. Altered synaptic pruning and synaptic connectivity have been proposed as mechanisms for both ASD and psychotic disorders (Germann et al., 2021; Van Spronsen et al., 2010; Pagani et al., 2021; Thomas et al., 2016; Cunningham et al., 2013). These mechanisms are not mutually exclusive and may act in concert or over different time courses to alter neurodevelopment. The precise physiological role of cytokines in the brain is not fully understood, but gain- and loss-of-function studies suggest these molecules can regulate key developmental processes. Our data showed unique profiles of pro- and anti-inflammatory cytokines in the STG, a region that is important in sensory and language processing across different mental health conditions. Individuals with ASD exhibited elevated levels of the IL-1 family of cytokines (IL-1α and IL-18), FLT-3L, M-CSF, G-CSF, IL-8, and GROα compared to both psychotic disorders and controls. In contrast, individuals with psychotic disorder display increased chemokines eotaxin, fractalkine and RANTES, and increased cytokines IL-15 and IL-12p70. Moreover, there were increased levels of the regulatory/anti-inflammatory cytokines IL-4, IL-10 and TGFβ1 in psychotic disorders relative to ASD and controls. In contrast individuals with ASD do not have this compensatory change in regulatory cytokines even in the presence of increased proinflammatory mediators. The impaired cytokine regulation in ASD may contribute to an immune environment dominated by inflammatory signals, whereas psychotic disorders show a more regulated immune response.

Elevated proinflammatory cytokine levels have been reported in other brain regions of individuals with ASD, including the cerebellum, anterior cingulate gyrus and frontal cortex (Li et al., 2009; Vargas et al., 2005; Wei et al., 2012). Furthermore, multiple transcriptomic studies of postmortem brain tissue consistently highlight activation of innate immunity/glial markers and cytokine signaling pathways in ASD (Hughes et al, 2023). Among these studies, elevated IL-1 levels in the serum, cerebrospinal fluid, and/or brains of individuals with ASD have consistently been reported (Masi et al., 2015, Vargas et al., 2005; Arenella et al., 2023; Ashwood et al., 2011; Hughes et al., 2022; Sreenivas et al., 2024).In addition, genetic defects in the IL-1 family of cytokines and receptors have been reported in ASD (Arenella et al., 2023; Allen-Brady et al., 2010; Piton et al., 2008). Among these are genes that encode the IL-1 receptor accessory protein-like 1 (IL1RAPL1) and IL-1 receptor accessory protein (IL-1RAcP) which are synapse-organizing proteins that mediate cell adhesion and recruitment of glutamatergic synaptic components (Choucair et al., 2015; Takahashi et al., 2013). Mice with *IL1RAPL1* deletion exhibit some ASD-like behaviors, as well as deficits in associative memory and cognition (Houbaert et al., 2013; Yasumura et al., 2013). The IL-1 family consists of 11 known members, including IL-1α, IL-1β, IL-1Ra and IL-18 (Gaballa et al., 2024). In this study both IL-1 and IL-18 were differentially increased in ASD brains. IL-1 and its receptors play important roles in neurological processes from early CNS development through adulthood, influencing neurogenesis, proliferation, migration, synapse formation and plasticity (Mantovani et al., 2019; Dinarello et al., 2018). IL-1 was the first cytokine identified with actions on the CNS, inducing fever and *de novo* production of IL-1 in the brain following peripheral of IL-1 injection (Mantovani et al., 2019, Besedovsky et al., 1986). In addition, IL-1 influences pain modulation, stress responses, and can induce behavioral changes after acute or repeated administration(Mantovani et al., 2019, Besedovsky et al., 1986, Bonaccorso et al., 2003). Elevated levels of IL-1 in the brain are linked with memory and cognitive impairments (Goshen et al., 2009, Viviani et al., 2003). Similarities between the IL-1 and IL-18 receptor complexes and signaling, and their abilities to modulate fever, stress, and behavioral responses, suggest that they mediate similar effects in ASD (Alboni et al., 2010).

Similar to our findings in STG brain tissue, increased levels of IL-8 are detected in serum, CSF, and brain tissues of individuals with ASD (Masi et al., 2015, Vargas et al., 2005, Ashwood et al., 2011, Singh et al., 2017, Pardo et al., 2017). Increased IL-8 levels have also been found in the CSF in depressive disorders (Runge et al., 2023; Kuzior et al., 2020; Runge et al., 2021). Furthermore, elevated serum IL-8 present within the first three weeks of life is predictive of poorer neurodevelopmental outcomes (Nist et al., 2019). Our data also showed that the proliferation survival factor FLT-3L and cytokine/growth factors M-CSF and G-CSF were increased in ASD brain. FLT-3L is important in dendritic cell (DC) function in the immune system and is increased in the CSF of individuals with ASD (Pardo et al., 2017). Previously, DC subsets were associated with abnormal amygdala volumes in children with ASD (Breece et al., 2013). These findings indicate activation of DC subsets in ASD may contribute to an amplified inflammatory response. Increased M-CSF can lead to microglia activation, microglia proliferation and release of IL-1 (Hao et al., 2002; Li et al., 2021). Increased M-CSF levels are negatively correlated with verbal ability in children with ASD (Rose & Ashwood, 2014). G-CSF levels are increased in plasma cord blood samples from ASD compared to unaffected sibling controls (Moreno et al., 2024) and remain elevated into early adolescence (Belica et al., 2023). G-CSF may be released due to brain injury as blocking G-CSF in maternal immune activation and gestational valproic acid exposure models rescue behaviors and reduces neuroinflammation in offspring (Durankus et al., 2022; Mishra et al., 2022).

Interestingly, IFNα2 and IL-4 levels are significantly lower in STG brain tissue of ASD compared to both psychotic disorder and control groups. The role of IFNα2 in neuroimmune processes or ASD has not been well described. In contrast, IL-4, primarily produced by T helper-2 (T_H_2) cells, is known to be dysregulated in ASD (Ashwood et al., 2011; Careaga et al., 2017; Nie et al., 2023; Rose et al., 2020). IL-4 plays a crucial role in neuroprotection following injury (Walsh et al., 2015) and regulates learning and memory (Derecki et al., 2010). IL-4 can shift microglia function, skewing to a phenotype that facilitates tissue repair, alleviates neuronal damage (He et al., 2020), and dampens astrocyte activation (Brodie et al., 1998). The reduction of IL-4 in ASD suggests a diminished capacity to regulate microglial activation and astrocyte responses, contributing to a prolonged neuroinflammatory state (Vargas et al., 2005). Lower levels of IL-4 in ASD may also reflect an imbalance that favors a more neuroinflammatory environment.

Of note, in STG brain tissue, TGFβ1 and IL-10 are elevated in patients with psychotic disorders but not ASD. These important anti-inflammatory cytokines have key roles in the control and regulation of immune responses, dampening ongoing inflammation, promoting tissue repair and restoration of homeostasis (Rose et al., 2020; Ashwood et al., 2008; Rose et al., 2018). IL-10 and TGFβ1 can act on microglia and astrocytes to shift these cells towards a less inflammatory phenotype, and has been shown to inhibit cytokine production and receptor expression in microglia (Sawada et al., 1999; Norden et al., 2014). IL-10 has also been shown to have involvement in synapse formation (Lim et al., 2013). In meta-analyses, TGFβ1 levels are elevated in the plasma in SCZ individuals but decreased in those with ASD (Pillinger et al., 2019, Masi et al., 2015), a pattern consistent with the findings from this study. Further, analyzing the ratios of inflammatory cytokines levels relative to TGFβ1 levels, highlights the skew towards an inflammatory profile and away from regulatory mechanisms in ASD compared to psychotic disorder or control groups. Overall, a distinct pattern emerged that the regulatory cytokines IL-4, IL-10, and TGFβ1 are increase in psychotic disorders but not in ASD.

In STG brain tissue, individuals with psychotic disorders differentially displayed elevated levels of chemokines including fractalkine (CXCL1), the only known ligand for the CX3CR1 receptor. In the brain, neurons and astrocytes can produce fractalkine but only microglia express CX3CR1, suggesting fractalkine signaling influences microglia preferentially (Mizutani et al., 2012). Pretreatment with fractalkine before an LPS stimulation dampens the inflammatory response of primary microglia, suggesting a potential protective role (Inoue et al., 2021). Increased levels of fractalkine in the brains from individuals with psychotic disorders may therefore represent an adaptive response to mitigate aberrant function due to damage or inflammation. Eotaxin, was increased in the psychotic disorder STG brain tissue and has previously been shown to be increased in the blood of patients with schizophrenia (Ermakov et al., 2023; Teixeira et al., 2008). Increased eotaxin is linked to impairments in cognitive functions and other features of schizophrenia, such as formal thought disorders (Ivanovska et al., 2020). A third chemokine, RANTES, was elevated in psychotic disorders but decreased in ASD. RANTES has neuroinflammatory roles in the context of traumatic brain injury, but little is known about its role in neurodevelopment (Mennicken et al., 1999; Dutta et al., 2019; Bedrossian et al., 2016; Lesh et al., 2018).

In addition to unique profiles of cytokines in ASD and psychotic disorders STG samples, there were also increased chemokine/growth factor mediators shared in both disorders compared to controls, suggesting altered pathways that are common to the pathophysiology of neurodevelopmental disorders. For example, FGF-2 promotes the proliferation of neural progenitor cells and astrocytes, influences neuronal function and brain growth, and can alter blood brain barrier (BBB) permeability (Reuss et al., 2003; van Scheltinga et al., 2010; Yoshimura et al., 2001). PDGF regulates multipotent progenitors that are distinct from neural stem cells (Kumari et al., 2020; Moore et al., 2013). MCP-3 is produced by astrocytes, acts as a differentiating factor for dopaminergic precursors and neurons as well as activate microglia following brain injury (Edman et al., 2008; Renner et al., 2011). Together, these neuroinflammatory mediators may play diverse roles in early brain development, synaptogenesis and neuronal network reorganization and, when disrupted, may contribute to ASD and psychotic disorders.

While this study provides valuable insights into neuroimmune differences in ASD and psychotic disorders, certain limitations should be considered. Postmortem brain studies inherently involve variability in factors such as cause of death, co-morbid conditions, postmortem interval (PMI), and medication history. Medication use, particularly antipsychotics in the psychotic disorder group, may have influenced cytokine levels, but prior studies suggested these medications do not fully account for observed differences (Chen et al., 2023; Li et al., 2022; Simsek et al., 2016)). Moreover, our relatively large sample size (n = 72) and rigorous tissue processing protocols help mitigate these concerns. In addition, this study did not include direct behavioral data linked to immune markers, but future work integrating clinical datasets, such as those from Autism BrainNet (SFARI) and the NIH NeuroBiobanks, could clarify associations between cytokine profiles and symptom severity. While most subjects were male, reflecting ASD prevalence, expanding female representation will be important for understanding potential sex-specific neuroimmune differences. Lastly, this study focused on the STG, a region implicated in both ASD and psychotic disorders, but investigating cytokine expression across multiple brain areas will provide a broader perspective on immune involvement in these conditions. Our future studies will expand on current findings to map cytokine levels to specific cell types in multiple brain regions, in relation to clinical characteristics across the human lifespan.

## Conclusions

Increased cytokine and chemokine levels were seen in the STG of individuals with ASD and psychotic disorders compared to controls. These profiles shared some similarities between disorders, but mostly the patterns were unique. ASD brains had increased inflammatory cytokine profiles but lacked a compensatory change in regulatory cytokines, suggesting a more pronounced shift towards neuroinflammation and away from homeostasis. Psychotic disorders had marked inflammatory chemokine levels but incorporated more anti-inflammatory mechanisms. These differences suggest that cytokine-mediated neuroinflammation may uniquely shape neurodevelopment in each condition. Cytokine changes could represent detrimental, adaptive, or neutral outcomes (or all three) depending on when and where they occur in the brain. Determining the relationship between elevated cytokines and the neuropathology of ASD and psychotic disorders requires careful evaluations at a region-specific manner over a lifespan. Further investigation into the timing of these immune changes and their potential as therapeutic targeting could provide valuable insights for developing novel treatment strategies for ASD and psychotic disorders.

## Supplementary Material

Tables

Tables are available in the Supplementary Files section.

Supplementary Files

This is a list of supplementary files associated with this preprint. Click to download.

- table148plexdatatables.pdf
- table248plexdatatables.pdf
- STGcohortinformationsuppTable1.docx
- tables2heatmap.pdf
- tables3heatmap.pdf

## Data Availability

The datasets used for the current statistical analysis are available from the corresponding author upon reasonable request.
